# Crystal structure of 1-[3,5-bis­(tri­fluoro­meth­yl)phen­yl]-2-bromo­ethan-1-one

**DOI:** 10.1107/S2056989018007478

**Published:** 2018-05-31

**Authors:** Sandeep Chandrashekharappa, Keshab M. Bairagi, Mahendra K. Mohan, Viresh Mohanlall, Kabange Kasumbwe, Katharigatta N. Venugopala, Susanta K. Nayak

**Affiliations:** a Institute for Stem Cell Biology and Regenerative Medicine (inStem), GKVK Campus, Bellary Road, Bangalore 560 065, Karnataka, India; b Department of Chemistry, Visvesvaraya National Institute of Technology, Nagpur 440010, Maharashtra, India; c Department of Biotechnology and Food Technology, Faculty of Applied Science, Durban University of Technology, Durban 4001, South Africa

**Keywords:** crystal structure, tri­fluoro­meth­yl)phenyl­bromo­ethanone, weak inter­actions

## Abstract

The title compound, C_10_H_5_BrF_6_O, synthesized *via* continuous stirring of 3,5-bis­(tri­fluoro­meth­yl) aceto­phenone with bromine in an acidic medium and concentrated under reduced pressure, crystallizes with four mol­ecules in the unit cell (*Z* = 4) and one formula unit in the asymmetric unit. In the crystal, mol­ecules are linked in a head-to-tail fashion into dimers along the *b-*axis direction through weak C—H⋯Br and C—O⋯C*sp* ^2^ inter­actions. C—H⋯O, C—F⋯π and F⋯F inter­actions are also observed.

## Abstract 2

The title compound crystallizes with four mol­ecules in the unit cell (Z = 4) and one formula unit in the asymmetric unit. In the crystal, mol­ecules are linked in a head-to-tail fashion into dimers along the b-axis direction through weak C—H⋯Br and C—O⋯C*sp* ^2^ inter­actions. C—H⋯O, C—F⋯π and F⋯F inter­actions are also observed

## Chemical context   

Substituted phenacyl bromides can be achieved by α-bromination of substituted ketones employing suitable bromination reagents such as mol­ecular bromine (Curran & Chang, 1989 ▸), copper bromide (King & Ostrum, 1964 ▸), *N*-bromo­succinimide (Tanemura *et al.*, 2004 ▸), 3-methyl­imidazolium tribromide (Chiappe *et al.*, 2004 ▸) and hydrogen bromide (Podgoršek *et al.*, 2009 ▸). In our previous communications, we tried to develop inter­mediates (Chopra *et al.*, 2007 ▸) for the construction of biologically active heterocyclic compounds (Kasumbwe *et al.*, 2017 ▸). In this context, the title compound serves as a synthetic precursor and finds application in the construction of pharmacologically active heterocyclic compounds (Venugopala *et al.*, 2018 ▸, 2007 ▸).
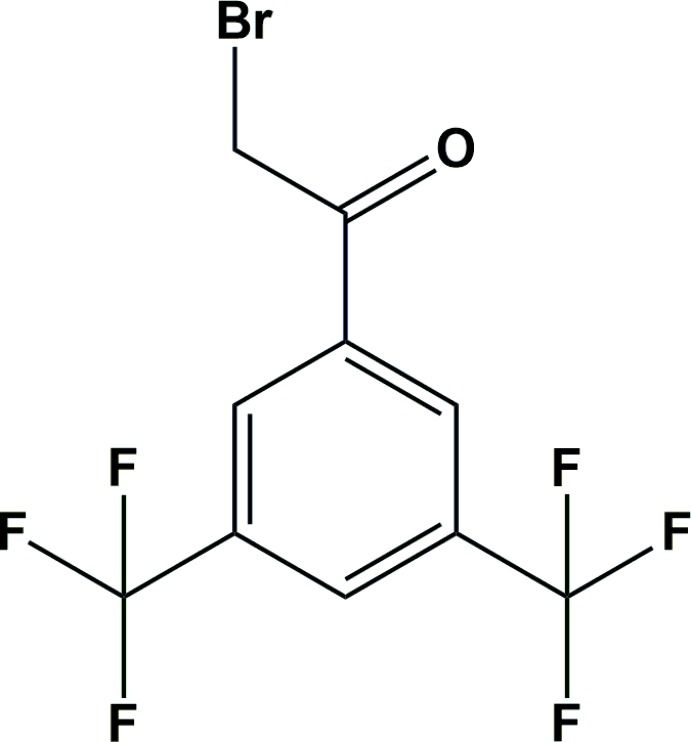


## Structural commentary   

**Figure 1:**
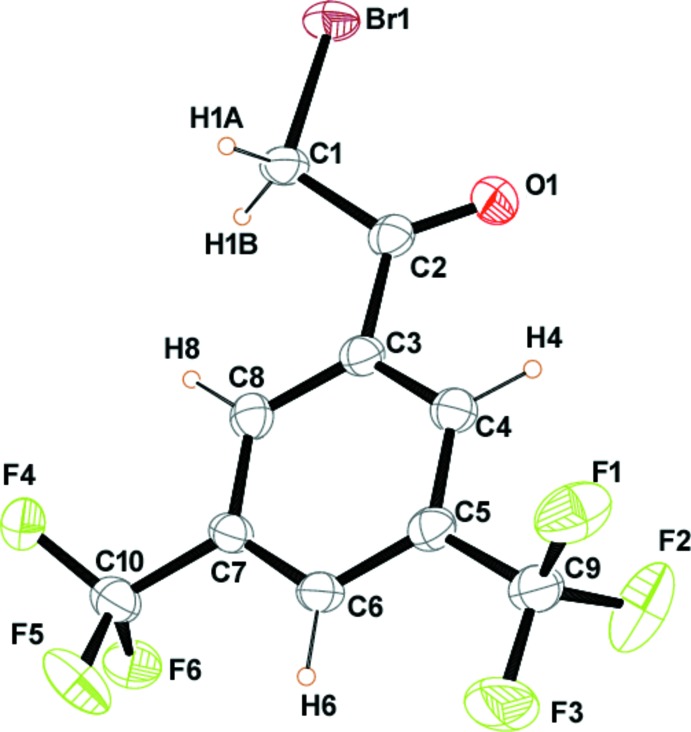
The asymmetric unit of the title compound, with 50% probability ellipsoids.

A displacement ellipsoid plot of the title compound with the atom labelling is shown in Fig. 1 ▸. The compound crystallizes in the monoclinic space group *P*2_1_/*c* with one mol­ecule in the asymmetric unit and four mol­ecules in the unit cell (*Z* = 4). The torsion angle between the alkyl bromide unit and the phenyl ring (C3—C2—C1—Br1) is −179.6 (3)° whereas that between the alkyl bromide and carbonyl parts (O1—C2—C1—Br1) is 0.3 (5)°, which shows a preference for a *syn* orientation of the alkyl bromide unit with respect to the carbonyl group.

## Supra­molecular features   

**Figure 2:**
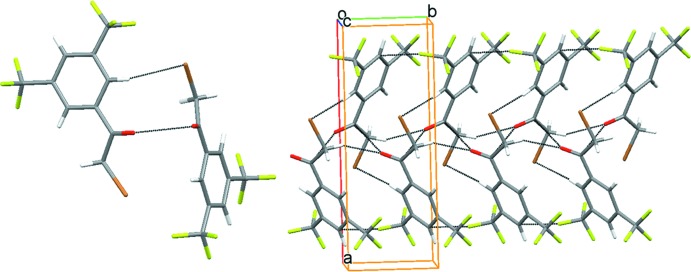
Dimer assembled through C—H⋯Br and >C=O⋯π(>C=O) inter­actions (left) and dimers extending along the *b-*axis direction *via* C—H⋯O and C—F⋯π inter­actions (Table 1 ▸).

**Figure 3:**
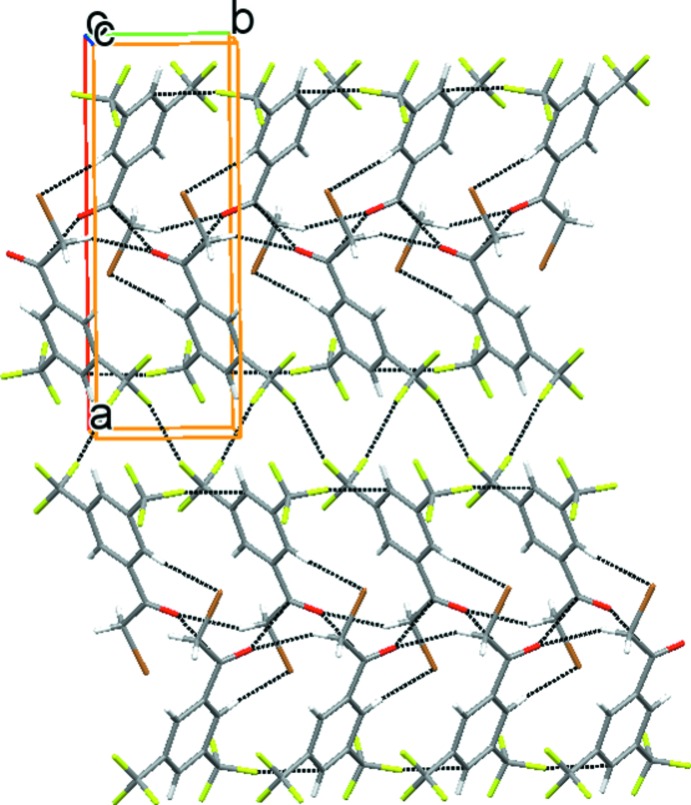
F⋯F inter­actions resulting in a bilayer that packs in a parallel fashion along the *a-*axis direction.

In the crystal, the mol­ecules are arranged in a head-to-tail fashion, forming dimers sustained by C—Br⋯H and >C=O⋯π(>C=O) (O⋯π = 3.252 Å) inter­actions. The dimers are linked along the *c*-axis direction by C—H⋯O and C—F⋯π inter­actions (Table 1 ▸, Fig. 2 ▸). The assembly of dimers is further extended along the *a*-axis direction by F1⋯F4(*x*, 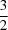 − *y*, 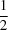 + *z*) [2.868 (4) Å] inter­actions, resulting in a bilayer which further packs in parallel fashion along the a-axis direction (Fig. 3 ▸).

## Database survey   

There are more than 1000 crystal structure of phenyl ethanone derivatives in the Cambridge Structural Database (CSD) (Conquest Version 1.17; Groom *et al.*, 2016 ▸) but none of them gave a hit for 1-[3,5-bis­(tri­fluoro­meth­yl)phen­yl]-2-bromo­ethanone. However, the crystal structures of related derivatives have been reported. These include phenyl 2-bromo­ethanone (URELEJ; Betz *et al.*, 2011 ▸) and a phenyl 2-bromo­ethanone complex (VIVFIP; Laube *et al.*, 1991 ▸). The first compound, *Z* = 4, features two prominent hydrogen bonds involving the oxygen atom while in the second, also *Z* = 4, the oxygen atom forms a complex with anti­mony penta­chloride.

## Synthesis and crystallization   

A stirred solution of 3,5-bis­(tri­fluoro­meth­yl) aceto­phenone (0.5 g, 1.95 mmol) in acetic acid (5 mL) was added dropwise to bromine (0.312 g, 1.95 mmol) in acetic acid. The reaction medium was stirred at room temperature for 5 h. To the resulting mixture, water (5 mL) was added and the mixture was concentrated under reduced pressure. The residue obtained was diluted with ethyl­acetate (10 mL), the organic layer washed with water (10 mL) and a sodium bicarbonate solution (5 mL), and filtered through dried sodium sulfate and evaporated to obtain 1-(3,5-bis­(tri­fluoro­meth­yl)phen­yl)-2-bromo­ethanone as a light-yellow solid in 62% yield. m.p: 317–318 K. ^1^H NMR: (CDCl_3_, 600 MHz): 8.44 (2H, *s*), 8.13 (1H, *s*), 4.48 (2H, *s*); ^13^C NMR: (CDCl_3_, 150 MHz): 188.81, 135.31, 133.06, 132.83, 132.60, 128.99, 127.08, 127.06, 125.42, 123.61, 121.80, 120.00, 29.46.

## Refinement   

Crystal data, data collection and structure refinement details are summarized in Table 2 ▸. Hydrogen atoms were placed in idealized positions (C—H = 0.95–0.99 Å) and refined using a riding model with *U*_iso_(H) = 1.2–1.5*U*_eq_(C).

## Supplementary Material

Crystal structure: contains datablock(s) global, I. DOI: 10.1107/S2056989018007478/ds2250sup1.cif

Structure factors: contains datablock(s) I. DOI: 10.1107/S2056989018007478/ds2250Isup2.hkl

Supporting information file. DOI: 10.1107/S2056989018007478/ds2250Isup3.cml

CCDC reference: 1843826

Additional supporting information:  crystallographic information; 3D view; checkCIF report

## Figures and Tables

**Table 1:** Hydrogen-bond geometry (Å, °)

*D*—H⋯*A*	*D*—H	H⋯*A*	*D*⋯*A*	*D*—H⋯*A*
C1—H1*A*⋯O1^i^	0.99	2.57	3.501 (5)	157
C1—Br1⋯H4^ii^	1.92 (1)	2.94 (11)	3.882	169
C2—O1⋯C2^iii^	1.20 (1)	3.05 (1)	4.126	149 (1)
C9—F2⋯π^iv^	1.32 (1)	3.89	4.848	130

Symmetry codes: (i) 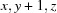; (ii) 
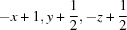
; (iii) 
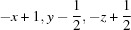
; (iv) 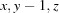.

**Table 2:** Experimental details

Crystal data	
Chemical formula	C_10_H_5_BrF_6_O
*M*_r_	335.04
Crystal system, space group	Monoclinic, *P*2_1_/*c*
Temperature (K)	153
*a*, *b*, *c* (Å)	14.156 (5), 5.0111 (16), 15.535 (5)
β (°)	104.316 (5)
*V* (Å^3^)	1067.7 (6)
*Z*	4
Radiation type	Mo *K*α
μ (mm^−1^)	3.92
Crystal size (mm)	0.23 × 0.09 × 0.06

Data collection	
Diffractometer	Bruker Kappa APEXII DUO
Absorption correction	Multi-scan (*SADABS*; Bruker, 2012 ▸)
*T*_min_, *T*_max_	0.442, 0.759
No. of measured, independent and observed [*I* > 2σ(*I*)] reflections	11628, 2405, 1741
*R*_int_	0.060
(sin θ/λ)_max_ (Å^−1^)	0.646

Refinement	
*R*[*F* ^2^ > 2σ(*F* ^2^)], *wR*(*F* ^2^), *S*	0.041, 0.103, 1.03
No. of reflections	2405
No. of parameters	163
H-atom treatment	H-atom parameters constrained
Δρ_max_, Δρ_min_ (e Å^−3^)	0.78, −1.12

Computer programs: *APEX2* and *SAINT* (Bruker, 2012 ▸), *SHELXS* (Sheldrick, 2008 ▸), *SHELXL2014* (Sheldrick, 2015 ▸), *Mercury* (Macrae *et al.*, 2008 ▸), *PLATON* (Spek, 2009 ▸) and *PARST* (Nardelli, 1995 ▸).

## supplementary crystallographic information

### Crystal data

**Table:**

C_10_H_5_BrF_6_O	*F*(000) = 648
*M**_r_* = 335.04	*D*_x_ = 2.084 Mg m^−^^3^
Monoclinic, *P*2_1_/*c*	Mo *K*α radiation, λ = 0.71073 Å
Hall symbol: -P 2ybc	Cell parameters from 2405 reflections
*a* = 14.156 (5) Å	θ = 2.7–27.4°
*b* = 5.0111 (16) Å	µ = 3.92 mm^−^^1^
*c* = 15.535 (5) Å	*T* = 153 K
β = 104.316 (5)°	Needle, colorless
*V* = 1067.7 (6) Å^3^	0.23 × 0.09 × 0.06 mm
*Z* = 4	

### Data collection

**Table:**

Bruker Kappa APEXII DUO diffractometer	2405 independent reflections
Radiation source: fine-focus sealed tube	1741 reflections with *I* > 2σ(*I*)
Graphite monochromator	*R*_int_ = 0.060
ω scans	θ_max_ = 27.4°, θ_min_ = 2.7°
Absorption correction: multi-scan (SADABS; Bruker, 2012)	*h* = −18→18
*T*_min_ = 0.442, *T*_max_ = 0.759	*k* = −6→6
11628 measured reflections	*l* = −20→20

### Refinement

**Table:**

Refinement on *F*^2^	Primary atom site location: structure-invariant direct methods
Least-squares matrix: full	Secondary atom site location: difference Fourier map
*R*[*F*^2^ > 2σ(*F*^2^)] = 0.041	Hydrogen site location: inferred from neighbouring sites
*wR*(*F*^2^) = 0.103	H-atom parameters constrained
*S* = 1.03	*w* = 1/[σ^2^(*F*_o_^2^) + (0.0541*P*)^2^ + 0.3605*P*] where *P* = (*F*_o_^2^ + 2*F*_c_^2^)/3
2405 reflections	(Δ/σ)_max_ < 0.001
163 parameters	Δρ_max_ = 0.78 e Å^−^^3^
0 restraints	Δρ_min_ = −1.12 e Å^−^^3^

### Special details

**Table:**

Geometry. All esds (except the esd in the dihedral angle between two l.s. planes) are estimated using the full covariance matrix. The cell esds are taken into account individually in the estimation of esds in distances, angles and torsion angles; correlations between esds in cell parameters are only used when they are defined by crystal symmetry. An approximate (isotropic) treatment of cell esds is used for estimating esds involving l.s. planes.

### Fractional atomic coordinates and isotropic or equivalent isotropic displacement parameters (Å^2^)

**Table:**

	*x*	*y*	*z*	*U*_iso_*/*U*_eq_	
Br1	0.60416 (2)	0.14882 (9)	0.09433 (3)	0.03259 (16)	
F1	0.21893 (17)	0.1990 (6)	0.38145 (15)	0.0468 (7)	
F2	0.1420 (2)	−0.0983 (6)	0.29661 (17)	0.0597 (8)	
F3	0.07527 (19)	0.2781 (7)	0.3050 (2)	0.0655 (9)	
F4	0.19332 (17)	0.8957 (5)	0.00403 (18)	0.0480 (7)	
F5	0.07651 (19)	0.8912 (5)	0.06942 (16)	0.0484 (7)	
F6	0.07736 (15)	0.6137 (5)	−0.03417 (14)	0.0362 (6)	
O1	0.45527 (18)	−0.0571 (6)	0.18612 (18)	0.0329 (6)	
C1	0.4825 (2)	0.3126 (8)	0.0979 (3)	0.0278 (9)	
H1A	0.4955	0.4921	0.1249	0.033*	
H1B	0.4423	0.3352	0.0365	0.033*	
C2	0.4258 (3)	0.1506 (8)	0.1506 (2)	0.0258 (8)	
C3	0.3301 (2)	0.2646 (8)	0.1557 (2)	0.0237 (8)	
C4	0.2889 (3)	0.1673 (8)	0.2217 (2)	0.0264 (8)	
H4	0.3218	0.0353	0.2620	0.032*	
C5	0.1991 (2)	0.2642 (8)	0.2285 (2)	0.0255 (8)	
C6	0.1483 (2)	0.4474 (8)	0.1686 (2)	0.0259 (8)	
H6	0.0857	0.5070	0.1722	0.031*	
C7	0.1901 (2)	0.5434 (8)	0.1028 (2)	0.0239 (8)	
C8	0.2806 (3)	0.4557 (8)	0.0968 (2)	0.0255 (8)	
H8	0.3091	0.5261	0.0524	0.031*	
C9	0.1583 (3)	0.1609 (9)	0.3022 (3)	0.0313 (9)	
C10	0.1348 (3)	0.7374 (9)	0.0362 (3)	0.0308 (9)	

### Atomic displacement parameters (Å^2^)

**Table:**

	*U*^11^	*U*^22^	*U*^33^	*U*^12^	*U*^13^	*U*^23^
Br1	0.0200 (2)	0.0386 (3)	0.0398 (3)	0.00282 (17)	0.00868 (15)	−0.0043 (2)
F1	0.0405 (14)	0.072 (2)	0.0306 (13)	−0.0144 (13)	0.0130 (11)	−0.0053 (12)
F2	0.093 (2)	0.044 (2)	0.0531 (17)	−0.0302 (15)	0.0394 (16)	−0.0108 (13)
F3	0.0370 (14)	0.094 (2)	0.077 (2)	0.0245 (15)	0.0356 (14)	0.0359 (17)
F4	0.0332 (13)	0.0366 (17)	0.0686 (18)	−0.0044 (11)	0.0020 (12)	0.0237 (13)
F5	0.0500 (14)	0.0464 (18)	0.0440 (14)	0.0282 (13)	0.0024 (11)	−0.0025 (12)
F6	0.0292 (11)	0.0432 (17)	0.0324 (12)	−0.0001 (10)	0.0005 (9)	−0.0029 (10)
O1	0.0285 (14)	0.0294 (18)	0.0420 (16)	0.0055 (12)	0.0109 (12)	0.0065 (13)
C1	0.0188 (16)	0.029 (3)	0.036 (2)	0.0010 (15)	0.0074 (15)	−0.0008 (17)
C2	0.0235 (17)	0.023 (2)	0.030 (2)	−0.0036 (16)	0.0046 (14)	−0.0040 (17)
C3	0.0203 (17)	0.023 (2)	0.028 (2)	−0.0016 (15)	0.0056 (15)	−0.0021 (16)
C4	0.0237 (17)	0.022 (2)	0.032 (2)	−0.0005 (15)	0.0052 (15)	−0.0020 (16)
C5	0.0230 (18)	0.025 (2)	0.028 (2)	−0.0036 (15)	0.0061 (15)	−0.0023 (16)
C6	0.0189 (16)	0.025 (2)	0.034 (2)	0.0007 (15)	0.0056 (15)	−0.0002 (17)
C7	0.0190 (16)	0.021 (2)	0.029 (2)	0.0009 (14)	0.0017 (14)	−0.0010 (16)
C8	0.0274 (18)	0.021 (2)	0.028 (2)	−0.0007 (15)	0.0071 (15)	−0.0012 (16)
C9	0.0253 (18)	0.032 (3)	0.037 (2)	−0.0022 (17)	0.0085 (16)	−0.0016 (18)
C10	0.0263 (19)	0.029 (2)	0.034 (2)	0.0032 (17)	0.0013 (16)	−0.0008 (18)

### Geometric parameters (Å, º)

**Table:**

Br1—C1	1.921 (4)	C3—C4	1.388 (5)
F1—C9	1.329 (4)	C3—C8	1.389 (5)
F2—C9	1.319 (5)	C4—C5	1.388 (5)
F3—C9	1.325 (4)	C4—H4	0.9500
F4—C10	1.330 (5)	C5—C6	1.377 (5)
F5—C10	1.324 (5)	C5—C9	1.497 (5)
F6—C10	1.343 (4)	C6—C7	1.388 (5)
O1—C2	1.203 (5)	C6—H6	0.9500
C1—C2	1.516 (5)	C7—C8	1.378 (5)
C1—H1A	0.9900	C7—C10	1.492 (5)
C1—H1B	0.9900	C8—H8	0.9500
C2—C3	1.490 (5)		
			
C2—C1—Br1	112.7 (3)	C7—C6—H6	120.6
C2—C1—H1A	109.1	C8—C7—C6	120.8 (3)
Br1—C1—H1A	109.1	C8—C7—C10	119.9 (3)
C2—C1—H1B	109.1	C6—C7—C10	119.3 (3)
Br1—C1—H1B	109.1	C7—C8—C3	120.1 (3)
H1A—C1—H1B	107.8	C7—C8—H8	119.9
O1—C2—C3	121.6 (3)	C3—C8—H8	119.9
O1—C2—C1	122.7 (3)	F2—C9—F3	107.2 (3)
C3—C2—C1	115.7 (3)	F2—C9—F1	105.4 (3)
C4—C3—C8	119.5 (3)	F3—C9—F1	106.3 (3)
C4—C3—C2	117.3 (3)	F2—C9—C5	112.7 (3)
C8—C3—C2	123.1 (3)	F3—C9—C5	112.7 (3)
C5—C4—C3	119.5 (4)	F1—C9—C5	112.1 (3)
C5—C4—H4	120.2	F5—C10—F4	107.8 (4)
C3—C4—H4	120.2	F5—C10—F6	106.0 (3)
C6—C5—C4	121.2 (3)	F4—C10—F6	106.1 (3)
C6—C5—C9	120.8 (3)	F5—C10—C7	112.4 (3)
C4—C5—C9	118.0 (4)	F4—C10—C7	112.4 (3)
C5—C6—C7	118.8 (3)	F6—C10—C7	111.8 (3)
C5—C6—H6	120.6		
			
Br1—C1—C2—O1	0.3 (5)	C10—C7—C8—C3	−176.6 (4)
Br1—C1—C2—C3	−179.6 (3)	C4—C3—C8—C7	−1.4 (6)
O1—C2—C3—C4	17.6 (5)	C2—C3—C8—C7	176.9 (3)
C1—C2—C3—C4	−162.5 (3)	C6—C5—C9—F2	117.1 (4)
O1—C2—C3—C8	−160.8 (4)	C4—C5—C9—F2	−62.1 (5)
C1—C2—C3—C8	19.1 (5)	C6—C5—C9—F3	−4.4 (6)
C8—C3—C4—C5	−0.6 (6)	C4—C5—C9—F3	176.5 (4)
C2—C3—C4—C5	−179.0 (3)	C6—C5—C9—F1	−124.2 (4)
C3—C4—C5—C6	2.7 (6)	C4—C5—C9—F1	56.6 (5)
C3—C4—C5—C9	−178.1 (3)	C8—C7—C10—F5	−151.0 (4)
C4—C5—C6—C7	−2.7 (6)	C6—C7—C10—F5	30.9 (5)
C9—C5—C6—C7	178.2 (3)	C8—C7—C10—F4	−29.3 (5)
C5—C6—C7—C8	0.5 (6)	C6—C7—C10—F4	152.6 (4)
C5—C6—C7—C10	178.7 (4)	C8—C7—C10—F6	89.9 (4)
C6—C7—C8—C3	1.5 (6)	C6—C7—C10—F6	−88.2 (4)

### Hydrogen-bond geometry (Å, º)

**Table:**

*D*—H···*A*	*D*—H	H···*A*	*D*···*A*	*D*—H···*A*
C1—H1*A*···O1^i^	0.99	2.57	3.501 (5)	157
C1—Br1···H4^ii^	1.92 (1)	2.94 (11)	3.882	169
C2—O1···C2^iii^	1.20 (1)	3.05 (1)	4.126	149 (1)
C9—F2···π^iv^	1.32 (1)	3.89	4.848	130

Symmetry codes: (i) *x*, *y*+1, *z*; (ii) −*x*+1, *y*+1/2, −*z*+1/2; (iii) −*x*+1, *y*−1/2, −*z*+1/2; (iv) *x*, *y*−1, *z*.
